# Unsuspected Leptospirosis Is a Cause of Acute Febrile Illness in Nicaragua

**DOI:** 10.1371/journal.pntd.0002941

**Published:** 2014-07-24

**Authors:** Megan E. Reller, Elsio A. Wunder, Jeremy J. Miles, Judith E. Flom, Orlando Mayorga, Christopher W. Woods, Albert I. Ko, J. Stephen Dumler, Armando J. Matute

**Affiliations:** 1 Division of Medical Microbiology, Department of Pathology, Johns Hopkins University School of Medicine, Baltimore, Maryland, United States of America; 2 Hubert-Yeargan Center for Global Health, Durham, North Carolina, United States of America; 3 Yale University Schools of Public Health and Medicine, New Haven, Connecticut, United States of America; 4 Duke University School of Medicine, Durham, North Carolina, United States of America; 5 Johns Hopkins Bloomberg School of Public Health, Baltimore, Maryland, United States of America; 6 Hospital Escuela Oscar Danilo Rosales Arguello, Universidad Nacional Autonoma de Nicaragua, León, Nicaragua; 7 Division of Infectious Diseases, Department of Medicine, Duke University School of Medicine, Durham, North Carolina, United States of America; 8 University of Maryland School of Medicine, Baltimore, Maryland, United States of America; Universidad Peruana Cayetano Heredia, Peru

## Abstract

**Background:**

Epidemic severe leptospirosis was recognized in Nicaragua in 1995, but unrecognized epidemic and endemic disease remains unstudied.

**Methodology/Principal Findings:**

To determine the burden of and risk factors associated with symptomatic leptospirosis in Nicaragua, we prospectively studied patients presenting with fever at a large teaching hospital. Epidemiologic and clinical features were systematically recorded, and paired sera tested by IgM-ELISA to identify patients with probable and possible acute leptospirosis. Microscopic Agglutination Test and PCR were used to confirm acute leptospirosis. Among 704 patients with paired sera tested by MAT, 44 had acute leptospirosis. Patients with acute leptospirosis were more likely to present during rainy months and to report rural residence and fresh water exposure. The sensitivity of clinical impression and acute-phase IgM detected by ELISA were poor.

**Conclusions/Significance:**

Leptospirosis is a common (6.3%) but unrecognized cause of acute febrile illness in Nicaragua. Rapid point-of-care tests to support early diagnosis and treatment as well as tests to support population-based studies to delineate the epidemiology, incidence, and clinical spectrum of leptospirosis, both ideally pathogen-based, are needed.

## Author Summary

Leptospirosis, transmitted by pathogenic species of the bacterium *Leptospira*, is distributed worldwide but infections due to unrecognized epidemic or endemic disease are under-appreciated. We prospectively studied patients ≥2 years of age who presented with acute febrile illness in Nicaragua and systematically collected detailed information about exposures and features of the illness as well as serum and blood to confirm acute infections. Among 704 patients with paired sera tested by MAT, we found acute leptospirosis in 6.3% (44/704). Patients with acute leptospirosis were more likely to present during rainy months and to report rural residence and fresh water exposure. Leptospirosis is a common (6.3%) but unrecognized cause of acute febrile illness in Nicaragua.

## Introduction

Leptospirosis is a zoonosis of worldwide distribution caused by pathogenic species of the spirochete *Leptospira.* The genetic diversity of *Leptospira* is increasingly recognized. Currently there are 9 species of known pathogenicity to humans or animals, 5 of unclear clinical significance [1]. Humans usually become infected with *Leptospira* through direct or indirect exposure to the urine of infected wild or domestic animals, which is common in less developed countries wherein humans and animals often live in close proximity and sanitation is poor [2]–[5]. The median incidence of leptospirosis in humans in such countries is as high as 975 per 100,000 population per year. Longer survival of leptospires in the environment in warm, humid conditions contributes to the higher incidence of leptospirosis in tropical versus temperate regions and to the peak in leptospirosis infections in the rainy season in tropical countries and during the summer in temperate regions [6]. In ever increasing recognition of its public health importance, leptospirosis has now been classified as an emerging or reemerging infectious disease by the Centers for Disease Control and Prevention and the World Health Organization. In recognition of the disproportionate impact on rural and urban residents living in resource-poor setting, the WHO now classifies leptospirosis as a neglected zoonotic disease.

In the past twenty years, large outbreaks of human leptospirosis associated with occupational [7] and recreational exposures [3], heavy seasonal rainfall [8], [9], and natural disasters have been detected in many countries, including in Nicaragua [10]–[13]. These outbreaks are often initially attributed to other pathogens and are recognized only after severe and/or unusual clinical illness prompts an epidemiologic investigation [8], [11], [14], [15]. The occurrence and extent of illness related to unrecognized epidemics or sustained transmission between epidemics often is underappreciated because of limited resources for surveillance and difficulties with diagnosis. However, rigorous prospective study of unselected patients with fever coupled with near complete convalescent follow-up identified leptospirosis as a leading but unsuspected cause of acute febrile illness in Sri Lanka [16], and leptospirosis could be of similar importance in Nicaragua.

The presence of leptospirosis in animal reservoirs in Nicaragua and elsewhere in Central America has been recognized for >50 years [17], [18]. The annual incidence of leptospirosis in Nicaragua has been estimated to be 23.3 per million; however, the actual rate is uncertain, since few cases are identified and confirmed [3], [6]. To determine the burden of and risk factors associated with unrecognized leptospirosis in Nicaragua, we conducted a prospective study of children and adults presenting acutely with fever to a large teaching hospital from a region and time without a recognized epidemic.

## Methods

### Ethics statement

Study doctors verified eligibility and willingness to return for a 2–4 week convalescent follow-up visit and obtained written informed consent from patients (≥18 years) or parents (<18 years), and assent if aged 12–17 years. The institutional review boards of Johns Hopkins University and Duke University Medical Center (USA) as well as Universidad Nacional Autonoma de Nicaragua, León (Nicaragua) approved the study.

### Setting and patients

We recruited patients in the emergency department and adult and pediatric wards of Hospital Escuela Oscar Danilo Rosales Arguello (HEODRA), the 400-bed primary public teaching hospital of Universidad Nacional Autonoma de Nicaragua in León, Nicaragua. Between August 2008 and May 2009, we enrolled consecutive febrile (≥38°C, tympanic) patients ≥1 month old without prior (within 1 week) trauma or hospitalization who presented during the day or early evening hours Monday through Saturday. Dedicated study doctors verified eligibility and willingness to return for follow-up and obtained written informed consent from patients (≥18 years) or parents (<18 years), and assent if 12–17 years. Study personnel recorded structured epidemiological and clinical data, including duration of illness and clinical provider's presumptive diagnosis, on a standardized form and then obtained specimens for on-site clinician-requested testing and off-site research-related testing. Patients returned for clinical and serologic follow-up 2 to 4 weeks later, or were visited at home if they did not return and could be located. Blood was centrifuged and sera frozen on site at −80°C.

### Samples

Serum and EDTA-anti-coagulated blood samples were stored promptly at −80°C. Samples were shipped on dry ice to and within the United States to diagnose acute leptospirosis infections.

### Serological screening by ELISA

Sera were tested for the presence of specific anti-leptospiral IgM antibodies by ELISA (Institut Viron Serion GmgH, Warburg, Germany) after removal of rheumatoid factor at Johns Hopkins University per the manufacturer's instructions.

The assay provided qualitative results — positive, negative, and equivocal (borderline positive/negative). Using a standard curve and evaluation table provided with the kit, optical density (OD) measurements were adjusted for plate-to-plate variation with a correction factor to yield quantitative results that correlated with titers. [19].

### Serological testing by Microscopic Agglutination Test (MAT)

We tested serum samples with the MAT to confirm the diagnosis of leptospirosis serologically at Yale University [20]. Twenty-five reference strains were used, which represented 6 pathogenic and one non-pathogenic species (Table S1). Patoc, a non-pathogenic strain, was used as a marker for possible infections with serovars not included in the panel. The presumptive infecting serogroup was determined based on the serovar against which highest agglutination titers were directed.

### Quantitative real-time PCR for leptospirosis

DNA was prepared from 1 mL of archived EDTA-anticoagulated blood with the automated QIAsymphony SP system (Qiagen Inc., Valencia, CA) at Johns Hopkins. Quantitative real-time PCR for leptospirosis was performed at Yale University using 5′ nuclease (TaqMan) assay and primers that amplified a sequence of *lipL32*, a gene that is exclusively present in pathogenic *Leptospira*, as has been described previously [21]. Duplicate samples detected within 40 PCR cycles and Sanger sequenced to confirm amplification of *lipL32* gene from *Leptospira* species were considered positives.

### Testing algorithm (Figure 1)

**Figure 1:**
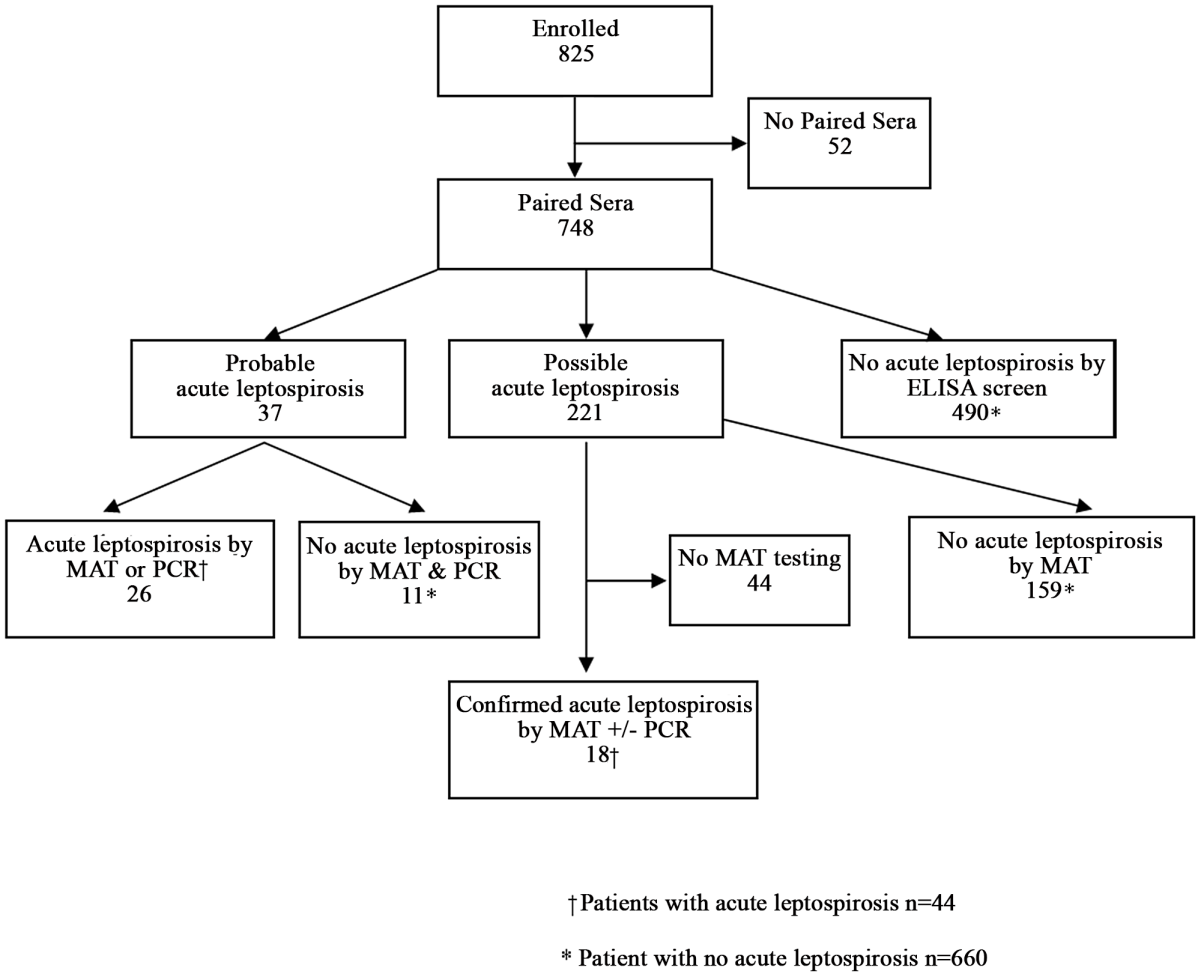
Study participants with a diagnosis of confirmed acute leptospirosis, Nicaragua 2008–9.

We tested paired sera by IgM ELISA to identify a subset of patients with probable and possible acute leptospirosis to be confirmed by MAT and/or PCR (Figure 1). In those with probable acute leptospirosis (IgM seroconversion by ELISA and/or the equivalent of a 4-fold rise in IgM titer), paired sera were tested by both MAT and PCR to confirm acute leptospirosis. In those with possible acute leptospirosis (stable, decreasing, or less than 4-fold rise in titer), paired sera were tested by MAT only if the convalescent-phase sera screened positive (titer 200 for a pathogenic serogroup) by MAT. In this latter group with possible acute leptospirosis, PCR was performed only for MAT-confirmed acute leptospirosis. Finally, PCR was performed in a subset of patients with unlikely acute leptospirosis (convalescent serum positive by ELISA but negative by MAT).

### Case definition

We defined a **confirmed case of acute leptospirosis** according to PCR and MAT criteria as proposed by the Center for Disease Control and Prevention in 2013 (http://wwwn.cdc.gov/NNDSS/). Briefly, in a patient with probable or possible acute leptospirosis identified by ELISA, confirmation required either a positive leptospirosis PCR result and/or seroconversion by MAT (defined as a negative acute-phase titer and convalescent-phase titer of ≥200, a 4-fold rise in titer by MAT between paired samples, or a single acute-phase MAT titer of ≥800).

### Statistical analysis

We compared proportions by the Chi-square test or Fisher's exact test and continuous variables by Student's t-test or the Wilcoxon rank sum test if not normally distributed. Confidence intervals for risk ratios were calculated by exact methods. We assessed IgM in the acute-phase sample for seroprevalence and clinical impression versus paired-sera testing for acute leptospirosis. We correlated epidemiologic features, duration of illness, and symptoms and signs with serologic results and performed univariable and multivariable logistic regression. We chose the multivariable model explaining the largest variance and with the lowest Akaike information criterion (AIC). Analyses were performed with Stata IC 11.0 (StataCorp LP, College Station, TX, USA).

## Results

### Patient characteristics

IgM ELISA testing for leptospirosis was completed for 800 (97.0%) of 825 consecutively enrolled patients. Of these 800, 748 (90.7%) had paired sera available, since 52 patients did not return and could not be located for follow-up. The likelihood of a subject returning for convalescent serum sampling and clinical follow-up did not differ by age (p = 0.90), sex (p = 0.93), or self-reported urban vs. rural residence (p = 0.53). The reported median distance from residence to hospital was 2 km (interquartile range [IQR] 2–20) for those who followed up versus 3 km (IQR 2–30) for those who did not (p = 0.08). Among the 748 patients with paired sera, the median age was 9 years (IQR 3–29). Slightly more were male (52.5%), and males were younger than females (median age 9 vs. 11, p = 0.007). The median reported duration of fever and of illness at presentation was 2 days and 3 days (IQR 1–4 and IQR 1–5, respectively). Many (30.0%) reported taking an antibiotic before presentation. The median interval between acute and convalescent follow-up was 15 days (IQR 14–28).

### Diagnosis of acute leptospirosis (Figure 1)

Testing paired sera by IgM ELISA identified 37 cases of probable acute leptospirosis, including 25 with seroconversion by ELISA (Figure 1). Notably, the acute-phase sera from all 25 cases with seroconversion by ELISA were also seronegative by MAT, as were an additional 2 acute-phase sera with equivocal IgM ELISA results from patients with paired ELISA results suggestive of a 4-fold rise in titer. Among the 37 cases of probable acute leptospirosis, 26 (70.3%) were confirmed (13 by MAT alone, 7 by MAT and PCR, and 6 by PCR alone) and 11 unconfirmed (no acute leptospirosis by MAT and PCR). Among the 20 (54.1%) of 37 cases of probable acute leptospirosis that were confirmed by MAT, 11 were seroconversions by ELISA. Among the 6 cases of probable acute leptospirosis confirmed by PCR alone, 5 were seroconversions by ELISA. Among the 221 cases of possible acute leptospirosis identified by ELISA, 177 had convalescent sera available for testing by MAT. Acute leptospirosis was ruled out by MAT in 159 of the 177 patients, of whom 30 were tested additionally by PCR and all 30 negative. However, acute leptospirosis was confirmed in 18 (10.2%) patients with possible acute leptospirosis (14 by MAT alone, 4 by MAT and PCR). Hence, screening by ELISA with confirmatory MAT and PCR identified a total of 44 (6.3%) with confirmed acute leptospirosis and excluded the diagnosis in 660 patients, with only 44 of the original 748 patients with paired sera not tested by MAT.

Clinicians suspected leptospirosis in only five (11.4%) patients with acute leptospirosis (sensitivity and specificity of clinical impression 11.4% [95% CI 3.8—24.6] and 99.7% [95% CI 98.9—100.0], respectively, with receiver operating characteristic curve (ROC) area 0.55 (95% CI 0.51–0.60). If a positive acute-phase IgM result were used to diagnose acute leptospirosis, 27 true infections would be detected (sensitivity 62.8%, 95% CI 46.7—77.0) but 137 patients would be diagnosed erroneously (specificity 77.0%, 95% CI 73.4–80.3, ROC area 0.699 [95% CI 0.62–0.77]). Among 20 probable and 18 possible cases of acute leptospirosis confirmed by MAT, only 3 were positive in the acute-phase sera (2 associated with a 4-fold rise in titer and 1 with stable high titer). In comparison, PCR on acute-phase blood specifically identified 7 of 20 ELISA-probable MAT-confirmed acute leptospirosis infections, an additional 6 ELISA-probable acute leptospirosis infections negative by MAT, and 4 of 18 ELISA-possible MAT-confirmed acute leptospirosis infections. All positives by PCR were confirmed to amplify the *lipL32* gene.

### Epidemiologic and clinical features of acute leptospirosis (Tables 1 and 2)

**Figure 2:**
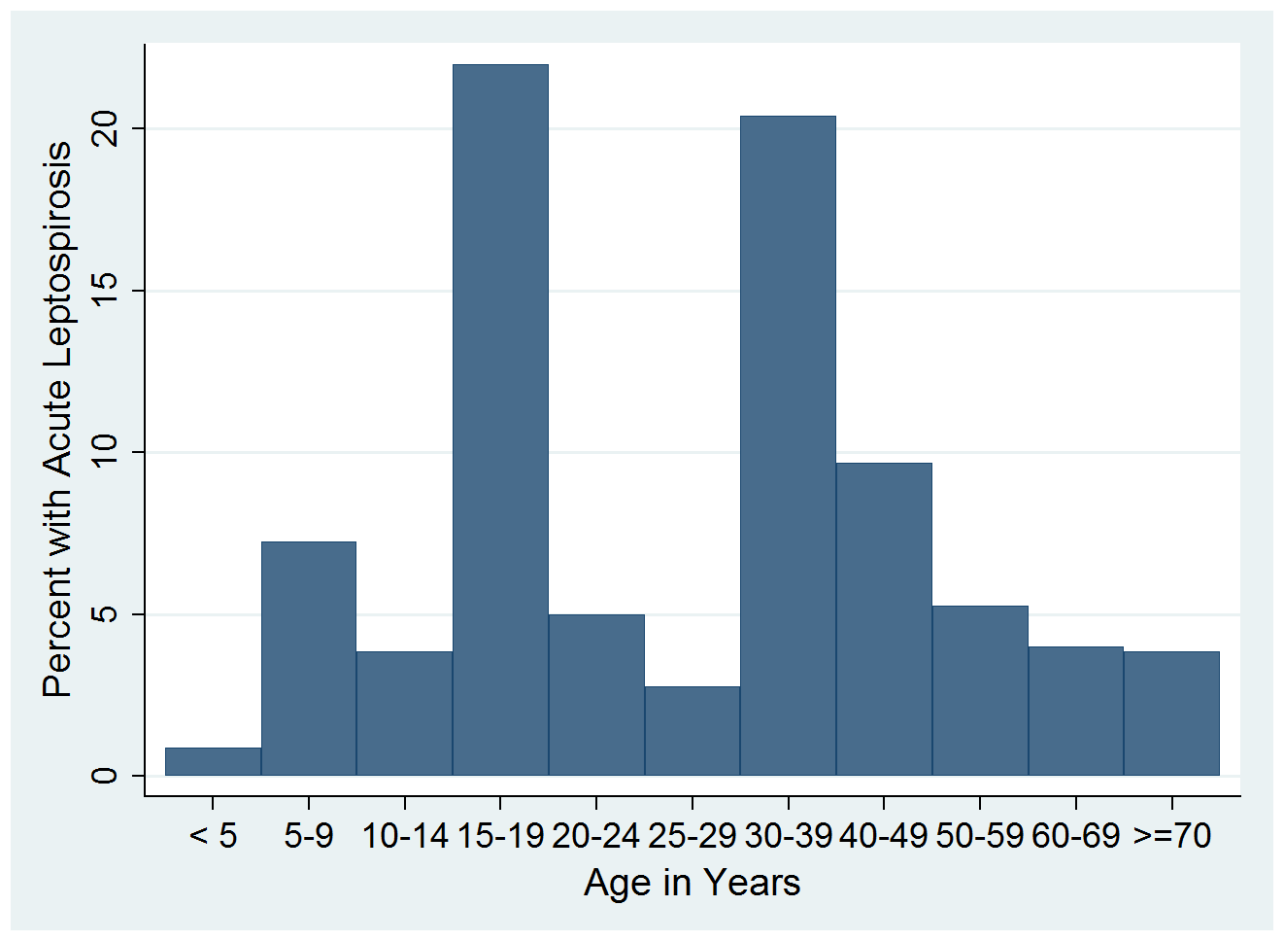
Age distribution of patients with confirmed acute leptospirosis, Nicaragua 2008–9.

**Figure 3:**
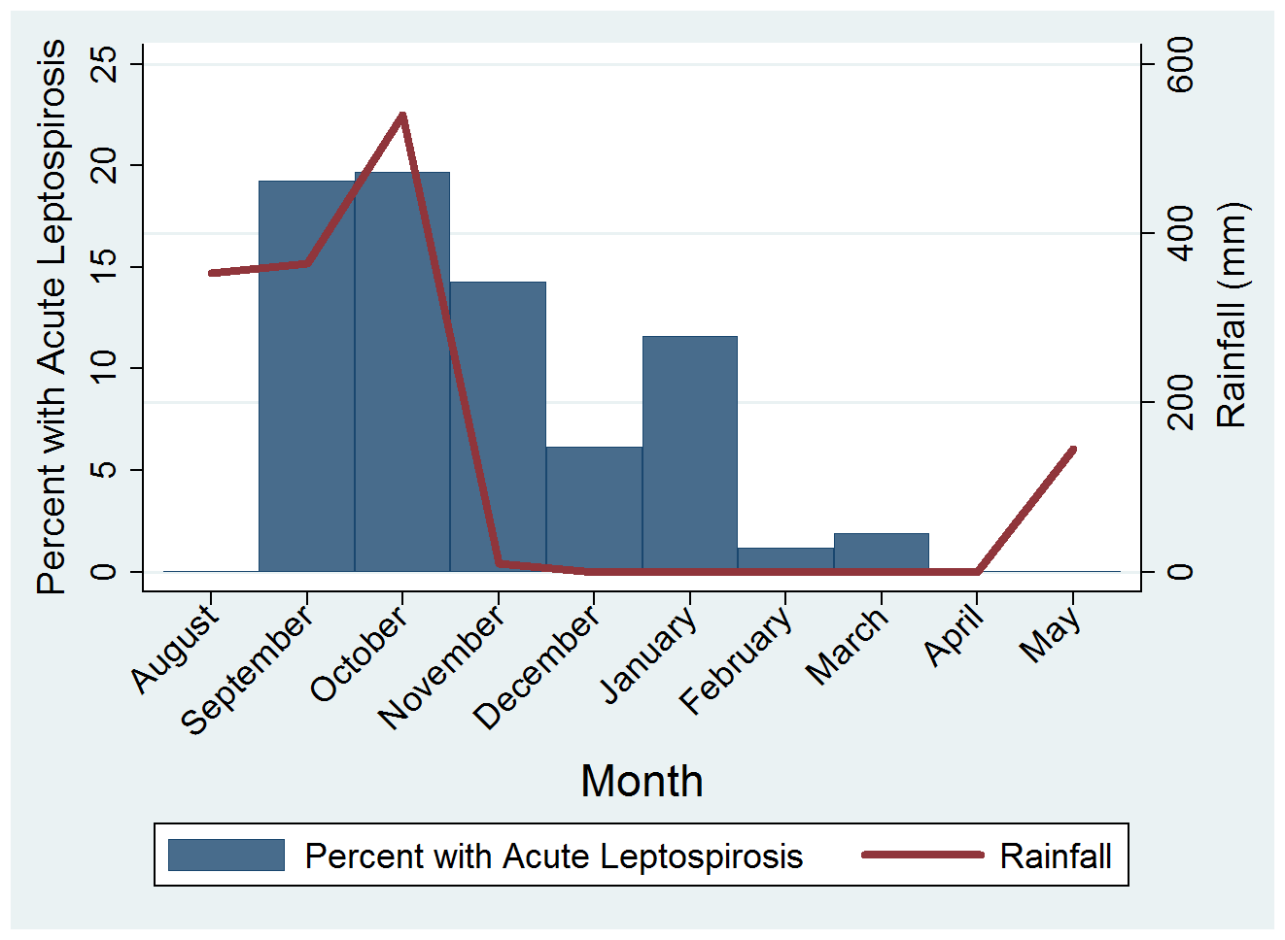
Acute leptospirosis and rainfall by month, Nicaragua 2008–9.

Epidemiologic and clinical characteristics of patients with and without acute leptospirosis are detailed in Tables 1 and 2, respectively. Those with and without acute leptospirosis had similar durations of illness (median 3.5 [IQR 2–6] vs. 3 [IQR 1–5] days, p = 0.10) and times to convalescent follow-up (14 days [IQR 14–31] versus 15 days [IQR 14–28], p = 0.73). Patients with acute leptospirosis were older (median 18 years, IQR 10–37) than febrile patients without acute leptospirosis (median 9 years, IQR 2–27), p = 0.0009 (Table 1 and Figure 2). The proportion of patients with leptospirosis that were male vs. female was similar (55% vs. 53%, respectively, p = 0.82). Acute leptospirosis was a more common cause of fever in rural than in urban residents (12.6% vs. 3.8%, p<0.001). Patients reporting pig exposure were both more likely to report rural residence (60% vs. 40%, p<0.001) and more likely to have confirmed acute leptospirosis (45% vs. 23%, p = 0.001). However, rural residence was associated with a statistically significant increased risk of acute leptospirosis even in the absence of reported pig exposure (10.2% vs. 3.2%, p = 0.003). Patients with acute leptospirosis were more likely to report fresh water exposure than were others (36.4 vs. 10.3%, p<0.001), which was predominantly river exposure (34.1% vs. 9.7%, p<0.001). Acute leptospirosis occurred throughout the year, but 11.3% of the febrile cohort had acute leptospirosis in rainy May to October (median rainfall 364.6 cm, IQR 145.0–539.8 cm) versus 4.8% of the cohort in drier November to April (median 0 cm, IQR 0–1.2 cm), p = 0.003. The largest number of acute leptospirosis cases occurred in October and November, when leptospirosis accounted for 20% (13/66) and 14% (10/70) cases of acute febrile illness, respectively (Figure 3). Fresh water exposure was also associated with acute leptospirosis (reported in 36.4% with leptospirosis vs. 10.33% without leptospirosis, p<0.001), and fresh water exposure was reported as commonly (by 11.4% of patients) in the wet season as in the dry season.

**Table 1:** Epidemiologic characteristics of febrile patients with acute leptospirosis versus no acute leptospirosis, Nicaragua, 2008–9.

Epidemiologic characteristic	Acute leptospirosis (n = 44)	No acute leptospirosis (n = 660)	P-value
Median age, years (IQR)	18 (10–36.5)	9 (2–27)	0.0009
Male sex	55%	53%	0.82
Residence			<0.001
Urban	43%	74%	
Rural	57%	26%	
Education, if age ≥18			
Illiterate	13%	15%	0.28
Primary	54%	39%	
Secondary	33%	36%	
University	0%	10%	
Type of work			0.05
Home	36%	55%	
Student	39%	31%	
Worker	9%	7%	
Farmer	5%	2%	
Merchant	9%	2%	
Other	2%#	3%	
Animal exposures#			
Rodent	70%	70%	0.98
Dog	57%	66%	0.24
Horse	20%	16%	0.41
Cow	20%	13%	0.16
Pig	45%	23%	0.001
Cat	25%	25%	0.96
Goat	5%	3%	0.67
Swim/bathe/wade			
River	34%	10%	<0.001
Other fresh water	5%	1%	0.047
Water source*			
Tap	59%	78%	0.004
Well	39%	20%	0.005
River	2%	1%	0.20
Bottled/boiled	0%	1%	0.56

# Does not sum to 100% secondary to rounding.

*Sums to greater than 100% secondary to multiple exposures.

**Table 2:** Clinical characteristics of febrile patients with acute leptospirosis versus no acute leptospirosis, Nicaragua, 2008–9.

Clinical characteristic	Acute leptospirosis (n = 44)	No acute leptospirosis (n = 660)	P-value
Symptom			
Headache	84%	47%	<0.001
Chills	86%	60%	<0.001
Sore throat	25%	29%	0.57
Cough	14%	41%	<0.001
Dyspnea	11%	13%	0.52
Joint pain	61%	26%	<0.001
Muscle pain	61%	29%	<0.001
Lethargy	2%	14%	0.03
Abdominal pain	30%	26%	0.61
Emesis	45%	36%	0.19
Diarrhea	14%	18%	0.47
Dysuria	20%	13%	0.15
Oliguria	14%	7%	0.14
Sign			
Mean temperature (°C)	38.8 (SD 0.65)	38.8 (SD 0.66)	0.91
Median heart rate (per minute)	97 (IQR 75, 110)	100 (IQR 62, 170)	0.24
Median respiratory rate (“ ”)	19 (IQR 17, 27)	23 (IQR 12, 68)	0.0003
Median SBP (mmHg)	100 (IQR 90 , 120)	100 (IQR 70, 160)	0.03
Altered mental status	0%	3%	0.33
Stiff neck	0%	1%	1.00
Conjunctival suffusion	0%	1%	1.00
Pharyngeal exudate	34%	29%	0.50
Lymphadenopathy	11%	22%	0.11
Jaundice	0%	1%	0.53
Lung crackles	2%	12%	0.045
Tender spleen	5%	0%	0.002
Tender liver	7%	2%	0.05
Hepatomegaly	2%	2%	0.94
Rash	0%	8%	0.05
Laboratory parameter	Median (IQR)	Median (IQR)	
WBC per µL	9700 (8700–16100)	11875 (8700–16100)	0.004
ANC per µL	7092 (5244–9923)	8483 (5695–12240)	0.18
ALC per µL	1826 (1300–2292)	2452 (1536–3741)	0.0026
Hemoglobin (g/dL)	12.2 (11.0–13.6)	11.8 (10.7–13.0)	0.29
Platelets x 1000 per µL	216 (195–286)	277 (220–350)	0.02

SBP, Systolic blood pressure; ANC, Absolute neutrophil count; ALC, Absolute lymphocyte count; SD, standard deviation; IQR, interquartile range.

Clinical features associated with acute leptospirosis are detailed in Table 2. The duration of reported fever was similar in patients with and without acute leptospirosis (median 2 days, p = 0.09), but chills were more frequently reported in those with acute leptospirosis (86 vs. 60%, p<0.001). Those with acute leptospirosis were more likely to report headache, joint pain, and muscle pain and less likely to report cough than other patients. Most (89.2% [33/37]) patients with acute leptospirosis and headache also reported chills. Nearly all (92.9% [26/28]) patients with acute leptospirosis and joint or muscle pain reported both, with joint pain alone and muscle pain alone equally infrequent (3.6% [1/28] for each). In contrast, there was less complete (73.7% [151/205]) overlap in patients without acute leptospirosis, with muscle pain alone (17.6% [36/205]) more common than joint pain alone (8.8% [18/205]) among patients reporting either symptom. Physical findings in patients with acute leptospirosis were largely similar to those without acute leptospirosis, and conjunctival suffusion was not observed. Patients with acute leptospirosis had lower leukocyte counts (median 9,700 vs. 11,875/µL, p = 0.004) with lower lymphocyte counts (median 1826, vs. median 2452/µL, p = 0.003). Hemoglobin concentrations were similar, but platelet counts lower in those with acute leptospirosis (median 216,000 vs. 277,000/µL, p = 0.02).

In a multivariable logistic regression model in which potentially statistically significant (p≤0.10) epidemiologic and clinical features in univariable analyses were eligible for inclusion, rainy season, rural residence, fresh water exposure, headache, joint pain, and absence of cough were independently associated with confirmed acute leptospirosis. In the final model including these variables, the odds of acute leptospirosis in the rainy vs. the dry season was 2.10 (95% CI 1.03, 4.31, p = 0.04), with rural vs. urban residence 2.14 (95% CI 1.05, 4.37, p = 0.04), and with exposure to freshwater vs. no such exposure 2.89 (95% CI 1.36, 6.15, p = 0.006). The presence of headache and joint pain conferred 2.86 (95% CI 1.15, 7.14, p = 0.02) higher odds and 2.52 (95% CI 1.22, 5.18, p = 0.01), respectively, than no such exposure. In contrast, the presence of cough was associated with a lower odds (OR 0.23) of acute leptospirosis (95% CI 0.09, 0.57, p = 0.002).

Of those with confirmed acute leptospirosis, 12 (28%) reported taking an antibiotic before presentation, including a second generation cephalosporin (8), third-generation cephalosporin (2); fluoroquinolone (1), and chloramphenicol (1). Patients with acute leptospirosis were as likely to be admitted to hospital as others but the length of stay was shorter (median 2 days, IQR 1.5–3.5 vs. 4 days, IQR 2–6, p = 0.008). Among 647 patients with data recorded, 10 (24.4%) of those with leptospirosis were treated with penicillin or amoxicillin vs. 187 (30.9%) of others (p = 0.38). No patient with leptospirosis received a tetracycline (vs. 1% of others, p = 0.60). Nearly all were asymptomatic at convalescent follow-up, similar (p = 0.11) to those without leptospirosis (80.1%). No one with leptospirosis died, but 9 of the 10 deaths in the study occurred before follow-up. Among those who died, the acute-phase serum was IgM-negative in 7, positive in 2, and equivocal in 1.

#### Presumptive agent

Confirmation of probable and possible cases by MAT suggested circulation of multiple serovars (Table S2), including Autumnalis, Ballum, Bataviae, Bratislava, Canicola, Copenhageni, Djasiman, Hebdomadis, Icterohamorrhagiae, Mini, Pomona, Pyrogenes, and Wolfii. Exposure to pigs was reported by 23.5% of patients, and was significantly (p<0.001) associated with MAT-detected antibodies against serogroups of leptospirosis that have been clearly epidemiologically linked to pigs, such as Bratislava and Pomona [6].

## Discussion

This study identifies leptospirosis as a major (6.3%) and clinically unrecognized cause of acute febrile illness in Nicaragua, and provides strong evidence for either sustained high-level transmission of leptospirosis or an unrecognized epidemic. Evidence that leptospirosis is perhaps endemic in the area includes the identification of new (acute) infections throughout the 10-month study period during both rainy and dry months.

The presence of leptospirosis in domestic and wild animal reservoirs was first documented in Nicaragua in 1962 [17], [18], but disease in humans was not evaluated. Agriculture accounts for 20% of Nicaragua's GDP and employs more than 29% of the workforce (data: USDA foreign agricultural service), so frequent human exposure to leptospires would be expected. However, the threat to human health was not recognized until 1995, when epidemic “hemorrhagic fever” without jaundice or renal manifestations was reported in rural Nicaragua, including the region surrounding our study area, following heavy rains and flooding and a comprehensive investigation implicated leptospirosis. Two months later a cross-sectional sero-survey identified IgM anti-*Leptospira* antibodies in 85 (15%) of 566 persons studied, but only 25 (29.4%) reported febrile illness during the preceding two months. Investigators concluded that the epidemic's attack rate was 15%, and that a large proportion of outbreak-related leptospirosis infections were asymptomatic. However, those sero-positive could have had leptospirosis before the epidemic, since IgM antibodies can persist for a year in 40% [18].

We hypothesized that both the importance of leptospirosis as a cause of acute febrile illness and the clinical spectrum of leptospirosis in Nicaragua were underappreciated. Because of a uniquely high (90%) rate of follow-up, we were able to identify acute infections and reliably distinguish them from past infections by testing paired sera. We identified acute infections in 6.3% (44) of the febrile cohort, which is likely a conservative estimate since most of our acute cases were seroconversions detected at a median of 15 days follow-up and 10% of patients seroconvert ≥30 days after onset of illness [22]. The impact of early treatment with a penicillin, recorded for over 30% of patients without acute leptospirosis, is unknown. Use of antimicrobial agents can alter the clinical course and or serologic response in patients with leptospirosis [23]. Further, we did not test all cases of possible leptospirosis by PCR, or those screen-negative by ELISA using MAT or PCR. However, it is unlikely that many cases were missed. First, the proportion of acute leptospirosis confirmed by MAT was much higher in those with probable vs. possible leptospirosis (54.1% vs. 10.2%) and in the former group only 6 were MAT-negative but PCR-positive. Second, all 30 MAT-negative ELISA-possible cases tested by PCR were negative.

By rigorous diagnosis of acute leptospirosis based on paired serology, we identified headache, chills, muscle pain, and joint pain as frequent and discriminatory symptoms in contrast to physical signs, and headache, joint pain, and absence of cough as independent predictors of acute leptospirosis in our final multivariable model. Headache, chills, and musculoskeletal pain were associated with epidemic acute leptospirosis in Nicaragua in 1995 [12]. Although WHO's recommended case definition includes headache and muscle pain, muscle pain and joint pain were highly correlated in our dataset; joint pain was retained in the final model because it better discriminated between acute leptospirosis versus other acute febrile illness (not the intended use of the WHO case definition). Conjunctival suffusion, often touted as a sensitive and specific feature, was not identified in our patients with acute leptospirosis. However, disease was relatively mild, as evidenced by absence of end-organ involvement (no oliguria, jaundice, hemorrhage, lung crackles, altered mental status, neck stiffness), relatively normal vital signs, and nearly normal complete blood counts. Although dyspnea, cough, and hemorrhage can be observed with acute leptospirosis, these features were infrequent in our patients in contrast with those with leptospirosis-associated pulmonary hemorrhage in Nicaragua in 1995 and more recently in Brazil [12], [15]. Although anicteric undifferentiated febrile illness with infreqent respiratory symptoms contrasts with the severe disease reported in some recent outbreaks [13], [24], it is not unexpected in early (median 3 days) leptospirosis in unselected febrile patients. In Peru, seroconversion was also associated with symptomatic but not severe illness [25].

We were also able to prospectively evaluate potential epidemiologic risk factors for infection suggested by retrospective studies, and found that rainy season, rural residence, and fresh water exposure (related to non-tap water source or swimming, bathing, or wading) were independent predictors of acute leptospirosis. Heavy seasonal rainfall has been associated in multiple studies with outbreaks of leptospirosis [8], [9]. Studies in Brazil and Peru found both urban slums and rural residence to be associated with increased risk of leptospirosis [9], [15], [25]–[27]. The 1995 Nicaragua epidemic case-control study found walking through creeks, household rodents, and ownership of dogs with titers ≥400 to *Leptospira* were independently associated with illness [12]. Similar to our finding that fewer patients with leptospirosis reported drinking water from a tap, the post-epidemic 1995 Nicaragua serosurvey identified indoor water source as independently associated with protection from infection [28].

Strengths of this study include the prospective study design, large sample size, inclusion of an unselected population with fever, near complete follow-up, epidemiologic and clinical correlation, and extensive testing to confirm acute leptospirosis. We prospectively assessed epidemiologic and clinical data and used objective criteria to sequentially enroll a large cohort of febrile patients six days a week to prevent recall bias and minimize selection bias. By obtaining both acute and convalescent sera in over 90%, we avoided misclassification of previously exposed individuals as current clinical cases, a frequent occurrence when only acute sera are tested. Further, those from whom paired sera were not available did not differ significantly from the population included. That patients were well on follow-up is consistent with the lack of late complications reported by others [29]. Limitations include the paucity of general laboratory data, such as liver function tests and serum chemistries; however, the disease spectrum suggests that the clinical utility of non-specific laboratory tests would be low. Further, this reflects routine clinical practice in the public sector in Nicaragua. Our estimate of leptospirosis could also be low if a wider array of serogroups and serovars is circulating than are detected by the ELISA and MAT panel used, but the ELISA detects a large number of serogroups and serovars and a broad MAT panel was used.

Confirmation of probable and possible cases by MAT allowed us to identify likely circulation of multiple serogroups, including Autumnalis, Ballum, Bataviae, Australis, Canicola, Djasiman, Hebdomadis, Icterohaemorrhagiae, Mini, Pomona, and Pyrogenes, and Sejroe in our population, in contrast to another setting such as an urban slum in Brazil. Confirmation of circulation of these serogroups would require culture for leptospirosis, which was not possible in this study. MAT reactivity against serogroups Ballum and Icterohamorrhagiae suggest exposure to rats (maintenance hosts for these serogroups and/or mice); rat exposure was very common (70%) in the population studied. MAT reactivity against Canicola was also observed, and might be expected with 60% of patients exposed to dogs. Notable was the significant association between pig exposure and leptospirosis and of reported pig exposure and antibodies against pig-associated serogroups of leptospires, such as Bataviae, Australis, and Pomona.

In conclusion, we found that leptospirosis accounted for 6.3% of acute febrile illness in Nicaragua and was more common during rainy months, in rural residents, and after exposure to freshwater. Consistent with mild non-specific acute febrile illness, leptospirosis was clinically not suspected. Clinical diagnosis, single acute-phase IgM ELISA, and single acute-phase MAT were poor predictors of confirmed acute leptospirosis with PCR reasonably sensitive and also specific. The lack of agreement between testing acute sera vs. paired sera [16] underscores the need for improved point-of-care, especially pathogen-based, diagnostics. The occurrence of incident (acute) leptospirosis throughout the 10 months of the study suggests that leptospirosis is endemic in the region surrounding León or that unrecognized epidemics occur. A population-based incidence study is needed to define the full spectrum of disease. Nonetheless, the high proportion of febrile patients with acute infection underscores the importance of public health interventions to reduce transmission, which should include education about risk factors for leptospirosis [7], such as exposure to fresh water, and control of animal reservoirs.

## Supporting Information

Alternative Language Abstract S1 Spanish translation of the Abstract.
(DOCX)

Table S1 *Leptospira* stains used for testing by the microagglutination test.
(DOCX)

Table S2 Serogroups of *Leptospira* suggested by microagglutination testing, Nicaragua, 2008–9.
(DOCX)
