# Supplementary material for: Unsuspected Leptospirosis Is a Cause of Acute Febrile Illness in Nicaragua
Source: PLoS Negl Trop Dis. 2014 Jul 24;8(7):e2941. doi: 10.1371/journal.pntd.0002941 (PMC4109853; doi:10.1371/journal.pntd.0002941)
Supplement: Alternative Language Abstract S1 — Spanish translation of the Abstract. (DOCX) [file pntd.0002941.s001.docx]

Antecedentes**.** Una epidemia de leptospirosis severa fue reconocida en Nicaragua en 1995, pero la enfermedad endémica y enfermedad epidémica no reconocida permanecen sin estudiar. Metodología / Principales resultados. Para determinar el impacto de los factores de riesgo asociados con la leptospirosis sintomática en Nicaragua, se estudió prospectivamente a pacientes con fiebre en un hospital universitario. Características epidemiológicas y clínicas fueron registradas sistemáticamente, y sueros pareados para IgM-ELISA para identificar pacientes con leptospirosis aguda probable y posible. Prueba de aglutinación microscópica y PCR fueron utilizados para confirmar la leptospirosis aguda. De los 704 pacientes con sueros pareados para MAT, 44 tenían leptospirosis aguda. Los pacientes con leptospirosis aguda fueron más propensos a presentarse durante la estación lluviosa y reportan la residencia rural y la exposición de agua fresca. La sensibilidad de la impresión clínica y de fase aguda para IgM detectados por ELISA fueron pobres. Conclusiones / Importancia. La leptospirosis es una causa frecuente (6,3%), de enfermedad febril aguda no reconocida en Nicaragua. Las pruebas rápidas en el punto de atención para apoyar el diagnóstico temprano y el tratamiento, así como pruebas de apoyo a los estudios basados ​​en la población para delinear la epidemiología, incidencia y espectro clínico de leptospirosis, ambos basados ​​ idealmente en patógeno, son necesarias.
