# Supplementary material for: Unsuspected Leptospirosis Is a Cause of Acute Febrile Illness in Nicaragua
Source: PLoS Negl Trop Dis. 2014 Jul 24;8(7):e2941. doi: 10.1371/journal.pntd.0002941 (PMC4109853; doi:10.1371/journal.pntd.0002941)
Supplement: Table S1 — Leptospira stains used for testing by the microagglutination test. (DOCX) [file pntd.0002941.s002.docx]

| ***Leptospira* Species** | **Serogroup** | **Serovar** | **Strain** |
| --- | --- | --- | --- |
| *L. biflexa* | Semaranga | Patoc | Patoc 1 |
| *L. borgpetersenii* | Ballum | Castellonis | Castellon 3 |
| *L. borgpetersenii* | Ballum | Ballum | Mus 127 |
| *L. borgpetersenii* | Mini | Mini | Sari |
| *L. borgpetersenii* | Tarassovi | Tarassovi | Perepelicin |
| *L. interrogans* | Australis | Bratislava | Jez Bratislava |
| *L. interrogans* | Autumnalis | Autumnalis | Akiyami A |
| *L. interrogans* | Bataviae | Batavie | Van Tienen |
| *L. interrogans* | Canicola | Canicola | H. Ultrecht IV |
| *L. interrogans* | Djasiman | Djasiman | Djasiman |
| *L. interrogans* | Hebdomadis | Hebdomadis | Hebdomadis |
| *L. interrogans* | Icterohaemorrhagiae | Copenhageni | M 20 |
| *L. interrogans* | Icterohaemorrhagiae | Icterohaemorrhagiae | RGA |
| *L. interrogans* | Pomona | Pomona | Pomona |
| *L. interrogans* | Pyrogenes | Pyrogenes | Salinem |
| *L. interrogans* | Sejroe | Hardjo | Hardjoprajitno |
| *L. interrogans* | Sejroe | Wolffi | 3705 |
| *L. kirschneri* | Cynopteri | Cynopteri | 3522C |
| *L. kirschneri* | Grippotyphosa | Grippotyphosa | Duyster |
| *L. noguchii* | Louisiana | Louisiana | LSU 1945 |
| *L. noguchii* | Panama | Panama | CZ 214 K |
| *L. interrogans* | Icterohaemorrhagiae | Copenhageni | Fiocruz L1-130 |
| *L. santarosai* | Shermani | Shermani | 1342 K |
| *L. weilii* | Celledoni | Celledoni | Celledoni |
| *L. weilii* | Javanica | Coxi | Cox |
