# Supplementary material for: Unsuspected Leptospirosis Is a Cause of Acute Febrile Illness in Nicaragua
Source: PLoS Negl Trop Dis. 2014 Jul 24;8(7):e2941. doi: 10.1371/journal.pntd.0002941 (PMC4109853; doi:10.1371/journal.pntd.0002941)
Supplement: Table S2 — Serogroups of Leptospira suggested by microagglutination testing, Nicaragua, 2008–9. (DOCX) [file pntd.0002941.s003.docx]

|  | |  | |  | | |  |
| --- | --- | --- | --- | --- | --- | --- | --- |
| **Patient** | **Acute MAT titer** | | **Acute serogroup** | | **Con* MAT titer** | **Con* serogroup** | |
| **1** | 0 | |  | | 400 | Autumnalis | |
| **2** | 400 | | Patoc | | 200 | Autumnalis | |
| **3** | 0 | |  | | 800 | Autumnalis | |
| **4** | 0 | |  | | 400 | Autumnalis | |
| **5** | 0 | |  | | 400 | Autumnalis | |
| **6** | 0 | |  | | 800 | Autumnalis/Mini | |
| **7** | 0 | |  | | 200 | Ballum | |
| **8** | 0 | |  | | 800 | Bataviae | |
| **9** | 0 | |  | | 1600 | Australis | |
| **10** | 0 | |  | | 400 | Australis | |
| **11** | 1600 | | Australis | | 1600 | Australis | |
| **12** | 0 | |  | | 200 | Australis | |
| **13** | 0 | |  | | 12800 | Canicola | |
| **14** | 0 | |  | | 12800 | Canicola/Icterohaemorrhagiae | |
| **15** | 200 | | Pyrogenes | | 12800 | Canicola/Pyrogenes | |
| **16** | 0 | |  | | 1600 | Icterohamorrhagiae | |
| **17** | 0 | |  | | 1600 | Djasiman | |
| **18** | 0 | |  | | 400 | Hebdomadis | |
| **19** | 0 | |  | | 1600 | Hebdomadis | |
| **20** | 0 | |  | | 1600 | Hebdomadis | |
| **21** | 0 | |  | | 400 | Icterohamorrhagiae | |
| **22** | 0 | |  | | 400 | Icterohamorrhagiae | |
| **23** | 0 | |  | | 400 | Icterohamorrhagiae | |
| **24** | 0 | |  | | 400 | Icterohamorrhagiae/Bataviae | |
| **25** | 0 | |  | | 800 | Icterohamorrhagiae/Sejroe | |
| **26** | 0 | |  | | 200 | Mix | |
| **27** | 0 | |  | | 6400 | Pomona | |
| **28** | 400 | | Patoc | | 1600 | Pomona | |
| **29** | 0 | |  | | 3200 | Pomona | |
| **30** | 0 | |  | | 6400 | Pomona | |
| **31** | 0 | |  | | 800 | Pomona | |
| **32** | 200 | | Patoc | | 400 | Pomona | |
| **33** | 0 | |  | | 400 | Pomona/Autumnalis | |
| **34** | 0 | |  | | 400 | Pomona/Pyrogenes | |
| **35** | 200 | | Pomona/Mini | | 800 | Pomona | |
| **36** | 0 | |  | | 1600 | Pyrogenes | |
| **37** | 0 | |  | | 200 | Sejroe | |
| **38** | 0 | |  | | 800 | Sejroe/Autumnalis | |

* Con= convalescent
